# Conjugated linoleic acid or omega 3 fatty acids increase mitochondrial biosynthesis and metabolism in skeletal muscle cells

**DOI:** 10.1186/1476-511X-11-142

**Published:** 2012-10-30

**Authors:** Roger A Vaughan, Randi Garcia-Smith, Marco Bisoffi, Carole A Conn, Kristina A Trujillo

**Affiliations:** 1 Department of Health, Exercise and Sports Science, University of New Mexico, 1 University Blvd, Albuquerque, NM, 87131, USA; 2 Department of Biochemistry and Molecular Biology, University of New Mexico Health Sciences Center, 1 University Blvd, Albuquerque, NM, 87131, USA; 3 Department of IFCE: Nutrition, University of New Mexico, 1 University Blvd, Albuquerque, NM, 87131, USA

**Keywords:** PGC-1α, Glycolysis, Oxidative metabolism, Polyunsaturated fatty acids (PUFA), Eicosapentaenoic acid, Docosahexaenoic acid, CLA

## Abstract

**Background:**

Polyunsaturated fatty acids are popular dietary supplements advertised to contribute to weight loss by increasing fat metabolism in liver, but the effects on overall muscle metabolism are less established. We evaluated the effects of conjugated linoleic acid (CLA) or combination omega 3 on metabolic characteristics in muscle cells.

**Methods:**

Human rhabdomyosarcoma cells were treated with either DMSO control, or CLA or combination omega 3 for 24 or 48 hours. RNA was determined using quantitative reverse transcriptase polymerase chain reaction (qRT-PCR). Mitochondrial content was determined using flow cytometry and immunohistochemistry. Metabolism was quantified by measuring extracellular acidification and oxygen consumption rates.

**Results:**

Omega 3 significantly induced metabolic genes as well as oxidative metabolism (oxygen consumption), glycolytic capacity (extracellular acidification), and metabolic rate compared with control. Both treatments significantly increased mitochondrial content.

**Conclusion:**

Omega 3 fatty acids appear to enhance glycolytic, oxidative, and total metabolism. Moreover, both omega 3 and CLA treatment significantly increase mitochondrial content compared with control.

## Background

Polyunsaturated fatty acids (PUFAs) play wide-ranging roles in cell metabolism, signaling and inflammation. Of these PUFAs, very long chain eicosapentaenoic acid (EPA) and docosahexaenoic acid (DHA) found principally in fish have key roles in metabolism and inflammation [1-18]. EPA has been shown to reduce triacylglyceride formation and improve blood lipid profiles through interactions with sterol-regulatory element binding protein-1c and liver X receptor alpha [19]. DHA has been shown to enhance lipid oxidation and insulin sensitivity in skeletal muscle through AMPK activation [14]. Combinations of omega 3 are commonly consumed, and have been shown to increase fat oxidation, reducing body weight, and prevent weight gain [1,2,4-9,11-15,17,18,20]. Moreover, treatment with combination omega 3 has been shown to triple the expression of genes encoding regulatory factors that control mitochondrial biogenesis and oxidative metabolism including peroxisome proliferator-activated receptor co-activator 1 alpha (PGC-1α) in white adipocytes [7]. Combination omega 3 can now be prescribed to lower triacylglycerides and is currently one of the most common over-the-counter dietary supplements [21].

Conjugated linoleic acid (CLA), a PUFA found in grass-fed beef among other sources also plays a role in lipid metabolism [18,22-28]. CLA, like fish oil, is a popular dietary supplement marketed for its role in enhancing fat metabolism. CLA is purported to have several physiological functions, including appetite suppression, increased fat mobilization, and increased fatty acid oxidation [18,23-25]. Recently, the *trans*-10,*cis*-12 CLA but not the *cis*-9,*trans*-11 CLA isomer was shown to significantly increase lipolysis in human adipocytes [23]. CLA was also shown to modify hormone sensitive lipase and perilipin expression, key components of fatty acid utilization [23]. Moreover, CLA is purported to reduce fatty acid synthesis in adipocytes, suggesting that CLA discourages fat deposition directly contributing to body composition [22,24]. Interestingly, rodents were shown to be resistant to diet-induced weight gain following treatment with CLA, and had increased lipid oxidation with reduced levels of plasma insulin [24]. Rodent models have also shown significant weight loss when treated with CLA [24,27]. In addition, treatment of rodents with CLA reduces weight as well as increases hepatic RNA expression associated with fatty acid oxidation [26].

Clinically, mitochondrial dysfunction is associated with reduced capacity for fatty acid oxidation and inversely related to incidence of type II diabetes and obesity [29-34]. PGC-1α, an essential stimulator of mitochondrial biosynthesis has been shown to increase fatty acid oxidation through induction of peroxisome proliferator-activated receptor alpha (PPARα) [35-40]. PGC-1α expression is inversely related to incidence of type II diabetes and obesity and reduced propensity for fatty acid oxidation [29-34]. Induction of PGC-1α has also been shown to heighten metabolic rate through increased expression of mitochondrial uncoupling proteins [33,39-41]. Irisin, a hormone released by skeletal muscle following exercise, is induced by PGC-1α expression and increases metabolic rate through uncoupling protein 1 induction [42].

The role of PUFAs such as omega 3 and CLA in glucose metabolism and cellular uptake is less understood. Induction of PGC-1α has been linked to increased glucose transport and insulin sensitivity through glucose transporter 4 (GLUT4) [43]. GLUT4 is an insulin dependent glucose transporter found almost exclusively in skeletal muscle and adipocytes. An increase in GLUT4 expression is evidence of increased glucose uptake and glycolytic reliance [44].

While there is evidence supporting a role for PUFAs in lipid metabolism in hepatocytes and adipocytes, there is limited evidence evaluating the effects of omega 3 fatty acids and CLA on human skeletal muscle cell metabolism. Because muscle cell metabolism can also play a significant role in body composition, we investigated the effects of a combination of docosahexaenoic acid and eicosapentaenoic acid (combination omega 3) and CLA on oxidative and glycolytic capacities and related gene expression, as well as mitochondrial biosynthesis in human muscle cells.

## Results

### Glycolytic metabolism

**Figure 1:**
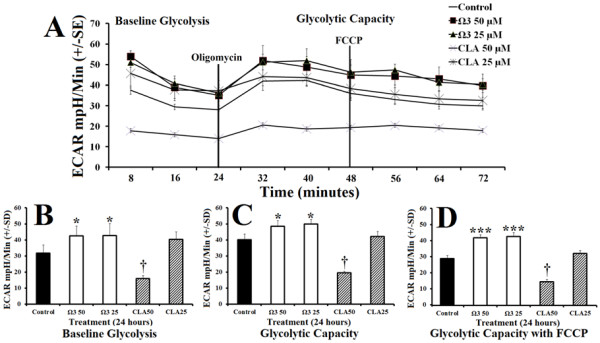
**Polyunsaturated fatty acids modify glycolytic metabolism. A**- Extracellular acidification rate (ECAR) of rhabdomyosarcoma cells treated with either DMSO control (0.1% final concentration), combination omega 3 (Ω3) at 25 μM or 50 μM, or CLA at 25 μM or 50 μM for 24 hours. **B**- Baseline ECAR following treatment described above. **C**- Peak ECAR following addition of oligomycin, an inhibitor of oxidative phosphorylation. **D**- Peak ECAR following addition of carbonyl cyanide p-[trifluoromethoxy]-phenyl-hydrazone (FCCP), a mitochondrial uncoupling agent, in addition to previously added oligomycin. *NOTES*: * indicates *p* < 0.05, ** indicates *p* < 0.01, and *** indicates *p* < 0.001 compared with control.

In order to examine effects of combination omega 3 or CLA treatment on glycolytic capacity in muscle cells, we measured extracellular acidification rate (ECAR) following treatment with either control, or combination omega 3 or CLA at 25 μM or 50 μM for 24 hours. ECAR was significantly elevated in cells treated with omega 3 at 25 μM or 50 μM for 24 hours compared with control (Figure 1A). Treatment with 25 μM CLA did not alter ECAR while treatment with 50 μM CLA significantly lowered ECAR (Figure 1A). Combination omega 3 treated cells exhibited a significantly greater ECAR compared with control (35% more than control) at baseline (Figure 1B). Combination omega 3 treated cells also demonstrated significantly higher total ECAR (27% more than control), a measure of glycolytic capacity induced by mitochondrial stress following addition of oligomycin (Figure 1C and D). *NOTE*: *FCCP was also added as an essential component of the oxidative stress kit and has no pronounced effect on glycolytic capacity*.

### Oxidative metabolism

**Figure 2:**
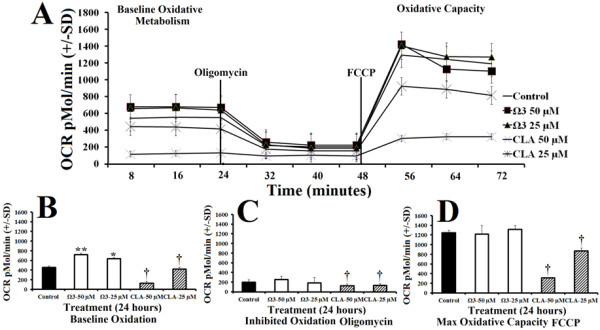
**Polyunsaturated fatty acids modify oxidative metabolism. A**- Oxygen consumption rate (OCR) of rhabdomyosarcoma cells treated with either DMSO control (0.1% final concentration), combination omega 3 (Ω3) at 25 μM or 50 μM, or CLA at 25 μM or 50 μM for 24 hours. **B**- Baseline OCR following treatment described above. **C**- OCR following addition of oligomycin, an inhibitor of oxidative phosphorylation. **D**- Peak OCR following addition of carbonyl cyanide p-[trifluoromethoxy]-phenyl-hydrazone (FCCP), a mitochondrial uncoupling agent, in addition to previously added oligomycin. *NOTES*: * indicates *p* < 0.05, ** indicates *p* < 0.01, and *** indicates *p* < 0.001 compared with control. † indicates *p* < 0.01 (significantly less than control).

To examine oxidative capacity, we measured oxygen consumption rate (OCR) following treatment with either control, or combination omega 3 or CLA at 25 μM or 50 μM for 24 hours. Oxygen consumption was significantly elevated 23% more than control in the omega 3 treated groups at baseline (Figure 2B). Omega 3 treatments did not significantly alter oxygen consumption following addition of oligomycin (an inhibitor of oxidative metabolism), or following the addition of FCCP (Figures 2C and D, respectively). Treatment with CLA decreased OCR in a dose dependent fashion during all stages of the metabolic stress (Figures 2A-D).

### Metabolic reliance

**Figure 3:**
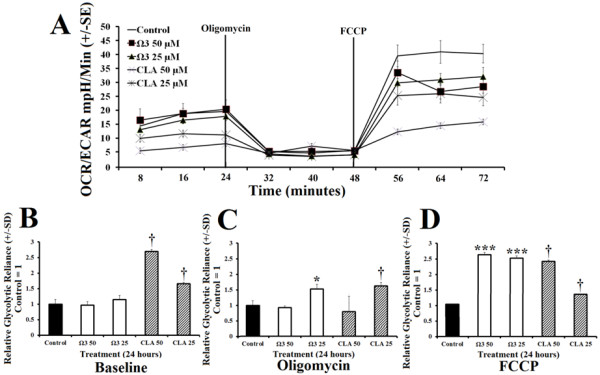
**Polyunsaturated fatty acids modify oxidative reliance OCR:ECAR. A**- Ratio of oxygen consumption rate (OCR) to extracellular acidification rate (ECAR) of rhabdomyosarcoma cells treated with either DMSO control (0.1% final concentration), combination omega 3 (Ω3) at 25 μM or 50 μM, or CLA at 25 μM or 50 μM for 24 hours. **B**- Relative glycolytic reliance at baseline OCR:ECAR following treatment described above with control normalized to value of 1. **C**- Relative glycolytic reliance from OCR:ECAR following addition of oligomycin (peak glycolysis), an inhibitor of oxidative phosphorylation. **D**- Relative glycolytic reliance with control = 1 from OCR:ECAR following addition of carbonyl cyanide p-[trifluoromethoxy]-phenyl-hydrazone (FCCP), a mitochondrial uncoupling agent, (peak oxidation) in addition to previously added oligomycin. *NOTES*: * indicates *p* < 0.05, ** indicates *p* < 0.01, and *** indicates *p* < 0.001 compared with control. † CLA had increased OCR:ECAR but had lower total individual OCR and ECAR compared with control.

Cellular reliance on glycolysis indicated by the ratio of OCR:ECAR, was significantly suppressed in omega 3 treated group compared with control (Figure 3A). Following oligomycin administration, omega 3 at 25 μM showed significantly greater reliance on glycolysis than the control (Figure 3C). After the addition of FCCP, treatment with omega 3 at 25 μM and 50 μM significantly increased cell reliance on glycolysis compared with control (Figure 3D). CLA treated groups exhibited an increased reliance on glycolysis but also showed significantly reduced total metabolism.

### Metabolic rate

**Figure 4:**
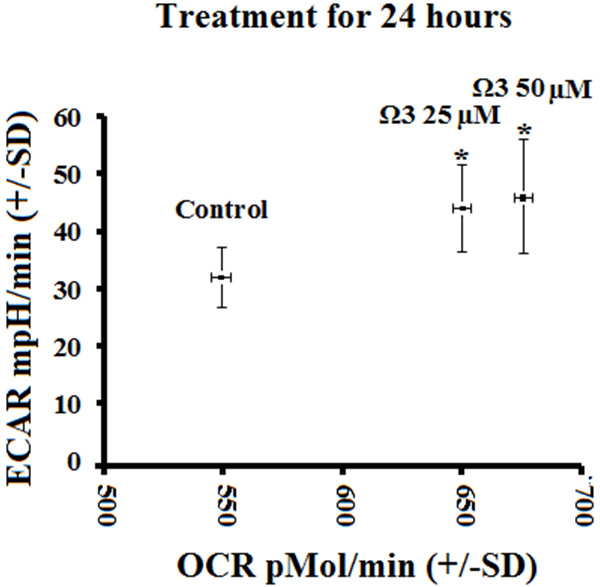
**Relative metabolic rate represented by oxygen consumption rate (OCR) versus extracellular acidification rate (ECAR) of rhabdomyosarcoma cells treated with either DMSO control (0.1% final concentration) or combination omega 3 (Ω3) at 25 μM or 50 μM for 24 hours. ***NOTES*: * indicates *p* < 0.05. Combination omega 3 fatty acids increase basal metabolic rate.

Combination omega 3 increased ECAR and OCR compared with control which indicates higher total metabolic rate (Figure 4). Treatment with either 25 or 50 μM combination omega 3 both significantly increased total metabolism compared with control, while CLA did not significantly increase metabolic rate (data not shown).

### Gene expression

**Figure 5:**
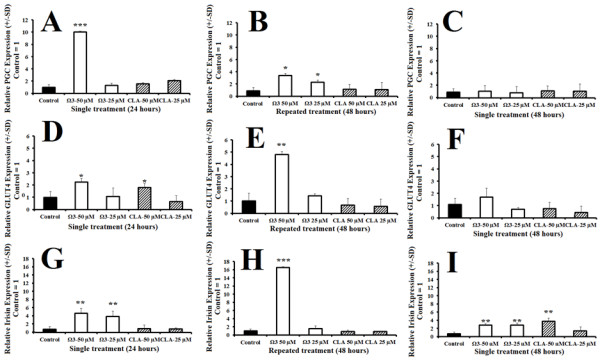
**Relative expression of PGC-1α (top row), GLUT4 (middle row) and Irisin (bottom row) following treatment with DMSO control (final concentration 0.1%), combination omega 3 at 50 μM, or CLA at 25 μM or 50 μM for either 24 hours (left), 48 hours with repeated treatment at 24 hours (center), or single treatment for 48 hours (right) with control = 1. A**- PGC-1α expression following treatment for 24 hours. **B**- PGC-1α expression following repeated treatment for 48 hours. **C**- PGC-1α expression following treatment for 48 hours. **D**- GLUT4 expression following treatment for 24 hours. **E**- GLUT4 expression following repeated treatment for 48 hours. **F**- GLUT4 expression following treatment for 48 hours. **G**- Irisin expression following treatment for 24 hours. **H**- Irisin expression following repeated treatment for 48 hours. **I**- Irisin expression following treatment for 48 hours. *NOTES*: * indicates *p* < 0.05, ** indicates *p* < 0.01, and *** indicates *p* < 0.001 compared with control. Polyunsaturated fatty acids modify metabolic gene expression.

To evaluate the effects of omega 3 or CLA treatment on select gene expression, we quantified relative RNA levels of PGC-1α, GLUT4, and irisin following treatment with either control, or combination omega 3 or CLA at 25 μM or 50 μM for 24 hours. Treatment with combination omega 3 at 50 μM for 24 hours significantly induced PGC-1α (Figure 5A). Treatment with combination omega 3 at 50 μM for 48 hours with a repeated treatment at 24 hours also significantly induced PGC-1α expression (Figure 5B). PGC-1α expression was returned to baseline at 48 hours following a single treatment of combination omega 3 at 50 μM (Figure 5C). GLUT4 was significantly induced by both treatments at 50 μM for 24 hours and by repeated treatment with combination omega 3 at 50 μM for 48 hours (Figure 5D and E), but was returned to baseline at 48 hours following a single treatment (Figure 5F). Irisin was significantly induced by both doses of combination omega 3 but not by either CLA treatment at 24 hours (Figure 5G). Treatment with omega 3 at 50 μM for 48 hours with repeated treatment significantly induced irisin (Figure 5H). Irisin was also elevated at 48 hours following a single treatment with either omega 3 and CLA (Figure 5I).

### Mitochondrial content

**Figure 6:**
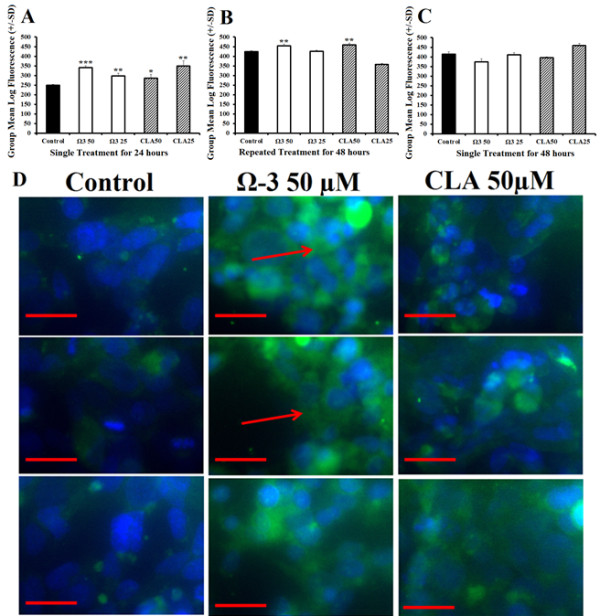
**Polyunsaturated fatty acids increase mitochondrial content. A**-Flow cytometry using mitochondrial staining of rhabdomyosarcoma cells treated with either DMSO control (0.1% final concentration), combination omega 3 (Ω3) at 25 μM or 50 μM, or CLA at 25 μM or 50 μM for 24 hours. **B**- Flow cytometry using mitochondrial staining following similar treatment described above for 48 hours with repeat treatment at 24 hours. **C**- Flow cytometry using mitochondrial staining following a single treatment described above for 48 hours. **D**- Immunohistochemistry of cells treated as described in Figure 4A and stained with Mitotracker (green) and DAPI (blue) with 0.1% DMSO control (left), Ω3 50 μM (middle) and CLA 50 μM (right). Red line indicates 50 μm and the red arrow indicates mitochondrial networking. *NOTES*: * indicates *p* < 0.05, ** indicates *p* < 0.01, and *** indicates *p* < 0.001 compared with control.

Treatment with either combination omega 3, or CLA at 25 μM or 50 μM for 24 hours significantly increased mitochondrial staining (Figure 6A). Cells treated with 50 μM omega 3 or CLA for 48 hours with a repeat treatment at 24 hours significantly increased mitochondrial staining (Figure 6B). Mitochondrial staining was returned to normal in cells treated with a single treatment of either combination omega 3, or CLA at 25 μM or 50 μM for 48 hours (Figure 6C). Following treatment described above, cells were stained with Mitotracker and DAPI and viewed for fluorescence. Microscopy revealed that cells treated with combination omega 3 or CLA consistently had greater fluorescence similar to flow cytometry results. Moreover, treated cells showed what appear to be greater number and size of mitochondrial networks (Figure 6D).

### Proliferation assay

**Figure 7:**
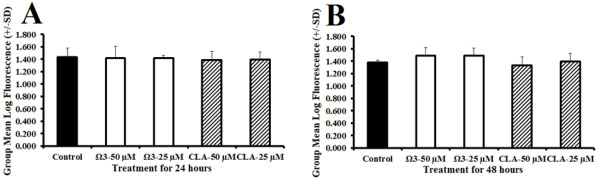
**Cell viability.** Measured by group mean log fluorescence from WST-1 end-point viability and proliferation assay following treatment of rhabdomyosarcoma cells with either DMSO control (0.1% final concentration), combination omega 3 (Ω3) at 25 μM or 50 μM, or CLA at 25 μM or 50 μM for 24 (**A**) or 48 hours (**B**).

Viability was assessed using WST-1 fluorescent proliferation assay which revealed no difference in cell viability and proliferation following treatment with omega 3, or CLA at 25 μM or 50 μM for 24 or 48 hours (Figure 7A and B).

## Discussion

Combination omega 3 significantly increased glycolytic capacity in muscle cells compared with control without suppressing oxidative metabolism suggesting that omega 3 increased total metabolisms (Figure 1B and 2B). Combination omega 3 significantly raised baseline oxygen consumption, a measure of oxidative metabolism and fatty acid oxidation as previously demonstrated [2,11,15-17,21]. Combination omega 3 also significantly decreased the ratio of OCR:ECAR suggesting that omega 3 fatty acids not only increase glycolytic capacity but also increase total glycolytic reliance. Treatment with CLA at 25 μM significantly decreased both glycolytic and oxidative metabolism. A decreased OCR:ECAR ratio suggests that, although total metabolism is suppressed, this treatment also induces a shift towards glycolytic metabolism. CLA at 50 μM did not alter ECAR, however OCR was reduced, also resulting in a lower OCR:ECAR ratio indicating a shift towards glycolytic metabolism.

The finding that maximum oxygen consumption is not increased in any treatment is interesting in light of the findings of increased mitochondrial content. First, following treatment for 24 and 48 hours, omega 3 significantly induced PGC-1α, an essential precursor for mitochondrial biosynthesis. This finding is supported by the increase in total mitochondrial content observed by both flow cytometry and microscopy. This suggests that both treatments are effective at increasing mitochondrial number, density and networking without influencing mitochondrial activity. Omega 3 treatment for 24 and 48 hours also significantly induced Irisin, a down-stream target of PGC-1α shown to enhance metabolic rate in rodents following exercise [42]. Moreover, omega 3 also increased GLUT4 expression, an insulin dependent glucose transporter exclusive to muscle cells and adipocytes, supporting the observation of increased glycolytic capacity.

The timing and duration of treatment played a significant role on mitochondrial changes. Treatment for 48 hours with repeated treatment at 24 hours also caused significantly greater mitochondrial staining compared with control. Remarkably, a single treatment for 48 hours has no significant effect on mitochondrial staining and a limited effect on gene expression; only Irisin expression was significantly greater than control following a single treatment for 48 hours. This observation supports the notion that while fish oils have many documented powerful effects, regular treatment may be necessary to sustain the potentially beneficial properties [2,11,15-17,21].

## Conclusion

Fish oil supplements and other polyunsaturated fatty acids including CLA are marketed heavily for their effects on metabolism. This work identified several effects that omega 3 fatty acids EPA and DHA as well as CLA (available over the counter to consumers) have on metabolism and mitochondrial characteristics in human muscle cells. Combination omega 3 and CLA increased the ratio of glycolytic metabolism to oxidative metabolism. However, with CLA treatment, the ratio is altered because of a decrease in oxidative metabolism rather than an increase in glycolytic metabolism, suggesting lower overall metabolism. We hypothesize that the clinical metabolic benefits of CLA consumption are due to the CLA-induced apoptosis of adipocytes in mammals, which liberates and increases fatty acid oxidation elsewhere in the body [45,46]. Omega 3 treatment significantly increased basal oxidative metabolism as well as basal and peak glycolytic metabolism. Because glycolytic metabolism is much less efficient, this shift likely results in greater glucose uptake. This is supported by up-regulation of the GLUT4 transporter. Based on these studies, combination omega 3 appears to be a potent stimulator of metabolism in muscle cells. More work is needed to identify the full capabilities of these bioactive lipids and the many other effects they likely have on metabolism.

## Materials and methods

### Cell culture and treatments

*Homo sapiens* rhabdomyosarcoma cells were purchased from ATCC (Manassas, VA). Cells were cultured in Dulbecco’s Modified Eagle’s Medium (DMEM) containing 4500mg/L glucose and supplemented with 10% heat-inactivated fetal bovine serum (FBS) and 100U/mL penicillin/streptomycin, in a humidified 5% CO_2_ atmosphere at 37°C. Trypsin-EDTA at 0.25% was used to detach the cells for splitting and re-culturing. All reagents were from Sigma (St. Louis, MO). Stock combination DHA:EPA with ratio of 1:2.5 (combination omega 3) or CLA from General Nutrition Center (Pittsburg, PA) was dissolved in DMSO to make treatment solutions; final concentration of DMSO 0.1% for all treatments. Cells were treated with either 25 μM or 50 μM omega 3 or 25 μM or 50 μM CLA and incubated for 24 or 48 hours (determined through pilot experiments) as described above.

### Quantitative real time polymerase chain reaction (qRT-PCR)

Cells were seeded overnight in 6-well plates at a density of 1 × 10^6^ cells/well and treated as described above. Following incubation, the total RNA was extracted using RNeasy Kit from Qiagen (Valencia, CA), per manufacturer’s protocol. Total RNA was quantified by Nanodrop spectrophotometry. cDNA was synthesized from 5000 ng total RNA using the Retroscript™ RT kit from Ambion (Austin, TX) according to manufacturer’s instructions. PCR primers were designed using Primer Express software from Invitrogen (Carlsbad, CA) and synthesized by Integrated DNA Technologies (Coralville, IA). Amplification of Irisin, GLUT4, and PGC-1α were normalized to the housekeeping gene, TATA Binding Protein (TBP). Table 1 summarizes the forward and reverse primers for TBP, Irisin, GLUT4, and PGC-1α. qRT-PCR reactions were performed in triplicate using the LightCycler 480 real-time PCR system from Roche Applied Science, (Indianapolis, IN). SYBR Green based PCR was performed in triplicate using 5000 ng of cDNA per sample; final primer concentrations were 10 μM in a total volume of 30 μl. The following cycling parameters were used: 95°C for 10 minutes followed by 45 cycles of 95°C for 15 seconds, and 60°C for one minute. Relative expression levels were determined by the ΔΔCp method and compared to the lowest expressing group [47].

**Table 1:** **Forward and reverse primer sequences used for qRT**-**PCR measurements synthesized by Integrated DNA Technologies** (**Coralville**, **IA**)

**Gene**	**Forward primer 5**^′^→ **3**^′^	**Reverse primer 5**^′^→ **3**^′^
PGC-1α	ACCAAACCCACAGAGAACAG	GGGTCAGAGGAAGAGATAAAGTTG
GLUT4	AAGAATCCCTGCAGCCTGGTAGAA	CCACGGCCAAACCACAACACATAA
Irisin	AGGTGCTTTACCGCTGTACCTTCA	AGAGAGGGCCAGATGTTTGTTGGA
TBP	CACGAACCACGGCACTGATT	TTTTCTTGCTGCCAGTCTGGAC

### Flow cytometry

Cells were plated into 6-well plates at a density of 1.2 × 10^6^ cells/well treated in triplicate and incubated as previously described for 24 or 48 hours. The cells were pelleted, the media was removed and the cells were suspended in pre-warmed media with 200 nM Mitotracker Green from Life Technologies (Carlsbad, CA) and incubated for 45 minutes (per manufactures’ protocol) and were incubated as previously described. The cells were pelleted, the media with Mitotracker was removed and the cells were suspended in pre-warmed media. Group mean fluorescence was measured using Facscalibur filtering at 488nm.

### Microscopy

Chamber-slides from BD Bioscience (Sparks, MD), were seeded with 5000 cells/well and treated in triplicate and incubated as previously described for 24 hours. The cells were then stained with either Mitotracker from Invitrogen (Grand Island, NY) for 45 minutes, and fixed in 3.7% formaldehyde in pre-warmed media. Cells were mounted with Prolong Gold with DAPI from Invitrogen (Carlsbad, CA) and cured overnight. Cells were imaged using the Axiovert 25 microscope with AxioCam MRc from Zeiss (Thornwood, NY).

### Metabolic assay

Cells were seeded overnight in 24-well culture plate from SeaHorse Bioscience (Billerica, MA) at density 5 × 10^5^ cells/well. Cells were treated and incubated for 24 hours as described above. Following treatment, culture media was removed and replaced with XF Assay Media from SeaHorse Bioscience (Billerica, MA) containing 4500mg/L glucose free of CO_2_ and incubated at 37°C. Per manufactures’ protocol, SeaHorse injection ports were loaded with oligomycin, and inhibitor of oxidative metabolism and maximizes glycolytic metabolism (final concentration 1.0 μM), carbonyl cyanide *p*-[trifluoromethoxy]-phenyl-hydrazone (FCCP), an uncoupler of electron transport maximizes oxidative metabolism (final concentration 1.25 μM), and rotenone in 1.0 μM final concentration. Extracellular acidification, a measure of glycolytic capacity, and oxygen consumption, a measure of oxidative metabolism was measured using the SeaHorse XF24 Extracellular Analyzer from SeaHorse Bioscience (Billerica, MA). SeaHorse XF24 Extracellular Analyzer was run using 8 minute cyclic protocol commands (mix for 3 minutes, let stand 2 minutes, and measure for 3 minutes) in triplicate.

### Proliferation assay

Cells were seeded in 96-well plates at density 5,000 cells/well and grown over night. Cells were treated and incubated as previously described for 24 or 48 hours. Media and treatment were removed at each time point and media containing 10% WST1 assay was added to each well and were incubated as previously described. Fluorescence was measured 1 hour following WST1 addition using Wallac Victor3V 1420 Multilabel Counter from PerkinElmer (Waltham, MA).

### Statistics

RNA gene expression, WST1 assay, cell metabolism, and flow cytometry were analyzed using ANOVA and pairwise comparisons comparing treatments with control. Values of *p* < 0.05 indicated statistical significance in all tests used and Bonferroni’s correction for error from multiple pairwise comparisons was used.

## Competing interests

The authors and contributors of this work report no conflict of interest.

## Authors’ contributions

RAV performed all experiments, was primary author of manuscript, produced experimental design, and performed statistical analyses. RG assisted in metabolic experiments. RAV, MB, CAC, and KAT assisted with experimental design and manuscript production. All authors read and approved the final manuscript.
